# An emerging master inducer and regulator for epithelial-mesenchymal transition and tumor metastasis: extracellular and intracellular ATP and its molecular functions and therapeutic potential

**DOI:** 10.1186/s12935-023-02859-0

**Published:** 2023-02-07

**Authors:** Yanyang Cao, Eileen Chen, Xuan Wang, Jingwen Song, Haiyun Zhang, Xiaozhuo Chen

**Affiliations:** 1 Department of Biological Sciences, Ohio University, Athens, OH USA; 2 Interdisciplinary Graduate Program in Molecular and Cellular Biology, Ohio University, Athens, OH USA; 3 The Edison Biotechnology Institute, Ohio University, Athens, OH USA; 4 Department of Chemistry and Biochemistry, Ohio University, Athens, OH USA; 5 Heritage College of Osteopathic Medicine, Ohio University, Athens, OH 45701 USA

**Keywords:** Tumor microenvironment, Invasion, EMT, ATP internalization, Macropinocytosis, Purinergic receptor

## Abstract

Despite the rapid development of therapeutic strategies in cancer treatment, metastasis remains the major cause of cancer-related death and scientific challenge. Epithelial-Mesenchymal Transition (EMT) plays a crucial role in cancer invasion and progression, a process by which tumor cells lose cell-cell adhesion and acquire increased invasiveness and metastatic activity. Recent work has uncovered some crucial roles of extracellular adenosine 5’- triphosphate (eATP), a major component of the tumor microenvironment (TME), in promoting tumor growth and metastasis. Intratumoral extracellular ATP (eATP), at levels of 100–700 µM, is 10^3^–10^4^ times higher than in normal tissues. In the current literature, eATP’s function in promoting metastasis has been relatively poorly understood as compared with intracellular ATP (iATP). Recent evidence has shown that cancer cells internalize eATP via macropinocytosis in vitro and in vivo, promoting cell growth and survival, drug resistance, and metastasis. Furthermore, ATP acts as a messenger molecule that activates P2 purinergic receptors expressed on both tumor and host cells, stimulating downstream signaling pathways to enhance the invasive and metastatic properties of tumor cells. Here, we review recent progress in understanding eATP’s role in each step of the metastatic cascade, including initiating invasion, inducing EMT, overcoming anoikis, facilitating intravasation, circulation, and extravasation, and eventually establishing metastatic colonization. Collectively, these studies reveal eATP’s important functions in many steps of metastasis and identify new opportunities for developing more effective therapeutic strategies to target ATP-associated processes in cancer.

## Background

Metastasis is the hallmark of malignant tumors and is responsible for about 90% of cancer mortality [1, 2]. Unlike localized primary tumors which can be treated with radiation, surgery, or chemotherapy, metastatic tumors are notoriously difficult to prevent, detect early, and treat due to genetic alteration or unique tumor microenvironment (TME). Thus, having a better understanding of the molecular mechanisms driving this process will be critical to developing effective novel strategies to target cancer metastasis and improve patient outcomes.

To achieve metastasis, tumor cells must migrate and separate from the primary tumor, invade surrounding normal tissues, circulate through the blood or lymphatic system, and colonize distant organs [2, 3]. Invasion of peritumoral tissue by traversing the basement membrane is critical to tumor progression and is considered to be the initial step of metastasis. During the course of invasion, carcinoma cells undergo epithelial-mesenchymal transition (EMT), lose intercellular connections, produce membrane filopodia-like protrusions as part of cytoskeleton remodeling, degrade and remodel the extracellular matrix (ECM), acquire invasive capabilities, and cross the basement membrane. Subsequently, cancer cells intravasate into the circulatory system, disseminate via blood or lymph vessels, extravasate, and eventually seed new tumor colonies in distant sites. Importantly, a large number of studies indicate that extracellular ATP (eATP) is involved in all of these steps of the metastatic cascade.

In this review, we will focus our discussion on the multiple mechanisms by which intratumoral eATP supports tumor cells in achieving metastatic colonization in distant organs, with emphasis on the earlier steps. These new findings suggest that elucidation of eATP’s roles in metastasis may lead to a better understanding of metastasis and novel therapeutic strategies for more effective anti-metastatic treatment.

## Epithelial-mesenchymal transition induction in metastasis

One of the initiating steps of primary tumor invasion is epithelial-mesenchymal transition (EMT), which is considered a crucial step in developing the invasive potential of cancer. During EMT, tumor cells lose epithelial cell-specific phenotypes and acquire mesenchymal traits that confer stem-like properties such as increased motility and invasive characteristics [4–7].

Tumor cells undergo EMT in response to a combination of extracellular cues in the TME. Stromal cells within the TME such as endothelial cells, fibroblasts, myofibroblasts, myeloid cells, and lymphoid cells all secrete a variety of soluble factors such as cytokines or growth factors, thereby inducing the EMT program in tumor cells. The summation of these multiple signaling pathways and regulators leads to the complex morphological, cellular, and gene expression changes during EMT. The major signaling pathways associated with EMT programs include TGF-β, Notch, Wnt, and growth factor receptor signaling. Among these, transforming growth factor (TGF-β) family signaling is thought to have a predominant role in EMT induction [8, 9]. TGF-β secreted by cancer cells or by the local stroma in the TME induces EMT, promotes cancer cell invasion and metastasis, and promotes drug resistance.

The EMT programs are coordinated by various EMT-associated transcription factors (EMT-TFs) as well as a series of microRNAs. EMT-TFs such as zinc-finger E-box-binding (ZEB), SNAIL, Slug, and TWIST families play a pivotal role in regulating gene expression during EMT progression [7, 10].

Importantly, recent evidence underscores the profound concept that EMT need not be considered an ‘all or none’ process [4, 11, 12], but an epithelial/mesenchymal (E/M) hybrid state. The mixed E/M phenotypes have been observed in many carcinoma cell lines including breast, lung, renal, and ovarian [13–16]. The current literature has demonstrated that the hybrid E/M state confers maximal tumor-initiating capacity and considers it a metastable phenotypic state [21–23]. In contrast, the carcinoma cells may have lost tumor-initiating and metastatic outgrowth capacity in a fully mesenchymal state [17, 19, 20]. The mixed E/M phenotypes are regulated by various transcription factors such as OVOL and GRLH2 that maintain the steady state [18]. The molecular mechanisms of stable hybrid E/M phenotypes are currently under intensive investigation and are not presently fully understood. However, the partial/hybrid EMT concept has been well established and supported by a wide variety of experimental evidence in many cancer types.

## Extracellular ATP and metastasis

### High-level ATP in the tumor microenvironment

Tumor growth, invasion, and metastasis depend on the bidirectional communication between tumor cells and their microenvironment. During cancer development, tumor cells release stimulatory growth factors, chemokines, and cytokines, which recruit stromal cells including infiltrating immune and vascular cells [21–23]. Likewise, these recruited cells release cytokines, growth factors, and proteases, as well as ECM proteins and basement membrane components [22, 24]. The key components of the TME include fibroblasts and myofibroblasts, immune and inflammatory cells, the extracellular matrix (ECM), and lymphatic and blood vessels [25, 26]. It is these cellular and biochemical components of the TME that are of prime importance in the regulation of cancer initiation, progression, and invasion.

Tumor cells utilize nutrients from the TME, such as glucose, glutamine, and essential amino acids, to sustain their uncontrolled proliferation [27, 28]. Surprisingly, the level of ATP has been shown to be greatly elevated in the TME. By using the novel engineered probe pmeLUC (plasma membrane luciferase) and bioluminescence imaging, Patrizia Pellegatti et al. have measured the extracellular ATP concentrations in vivo. They found that the ATP levels in the tumor interstitium are in the hundreds micromolar range, which are significantly higher than the interstitial ATP levels in healthy tissues (less than hundreds of Nanomoles per liter) [29].

There are multiple sources and mechanisms by which intratumoral eATP accumulates in the TME. The TME is characterized by heterogeneity in oxygenation, resulting in hypoxia-induced cell stress, injury, and death which all lead to ATP release [29, 30]. Other conditions such as the effect of chemotherapeutic agents, cell membrane damage, and autophagy can also cause the release of ATP into the extracellular space.

In addition to cell lytic ATP release, actively released ATP from active or apoptotic cells via vesicle exocytosis, transporters, or membrane-bounded channels also contribute to ATP accumulation in the TME. For example, exocytosis pathways, which secrete ATP from intracellular vesicles into the extracellular space, have been observed in various cell types including immune cells (e.g. T lymphocytes), platelets, and endothelial cells [31, 32].

Besides vesicular release, ABC transporters such as cystic fibrosis transmembrane conductance regulator (CFTR), multidrug resistance (MDR) gene product (also known as P-glycoprotein), and sulfonylurea receptor (SUR), are responsible for physically transporting ATP out of the cell for autocrine/paracrine purinergic signaling [31, 32].

**Fig. 1:**
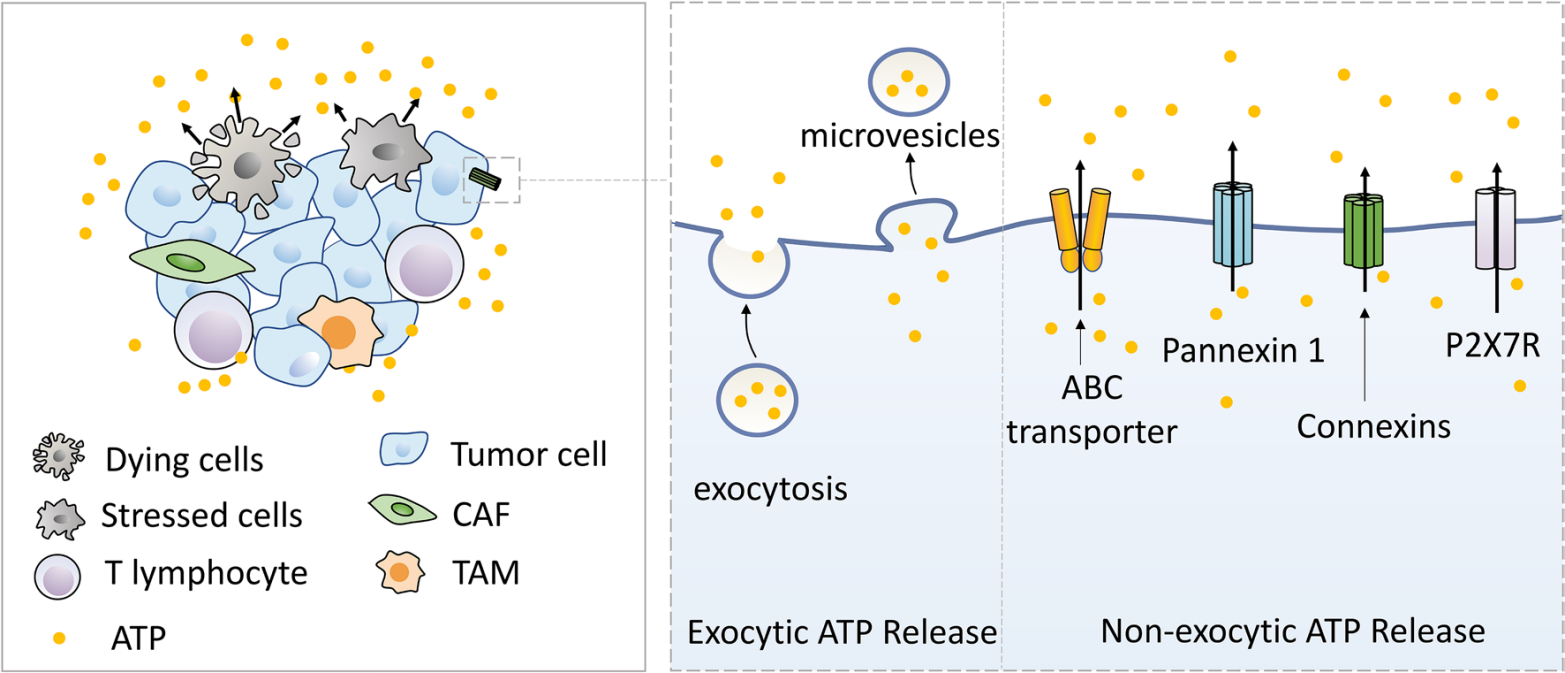
Mechanisms of ATP release into the tumor microenvironment. In cell lytic ATP-release (left), passively released ATP from stressed, injured, or dying tumor cells are the primary sources of intratumoral extracellular ATP in the TME. In addition, ATP actively released from active or apoptotic cells also contributes to ATP accumulation in the TME (right). Specifically, ATP release via microvesicles or vesicle exocytosis is involved in exocytic ATP release. ATP-binding cassette (ABC) transporters, connexin pannexin1, connexin channels, and the P2X7 receptor are involved in the release of non-exocytic ATP

Finally, intercellular channels including maxi-anion channels and pore-forming channels such as connexins, pannexins, and P2X purinergic receptor 7 (P2X7R), are also involved in ATP secretion. For instance, in many cell types, the primary mechanism for ATP release into the TME is considered to be plasma membrane proteins forming non-selective pores, such as the Pannexin 1 (Panx1) gap junction. In response to various stimuli such as hypoxia or apoptosis, Panx1 has been reported to form a hexamer channel which provides an efflux pathway for ATP release into the pericellular space [33, 34]. In fact, Panx1 regulates ATP release by a negative feedback mechanism involving the P2X7R, thus preventing potentially dangerous ATP accumulation in the extracellular environment [32, 35]. Specifically, Panx1 channels are closely associated with the P2X7R, where the ATP binding affinity for Panx1 is lower than P2X7R. Thus, as the eATP levels increase, ATP will first bind to P2X7R and inhibit the flux activity through Panx1 [36]. In summary, ATP can be released into the TME not only from dying or stressed cells but by living cells as well via various mechanisms. These mechanisms of ATP release are summarized in Fig. 1.

### Internalization of intratumoral ATP by macropinocytosis in cancer

Tumor cells alter their metabolism to support rapid proliferation and expansion in the body [37]. For example, cancer cells accelerate ATP production by dramatically increasing the rate of glycolysis in the cytosol instead of through the citric acid cycle and oxidative phosphorylation in the mitochondria [38, 39]. Consequently, the intracellular ATP (iATP) levels of tumor cells are higher than those in normal cells of the same tissue origin [40, 41].The increased rate of nutrient consumption and inadequate vascular supply within a tumor result in nutrient scarcity. In response to this suboptimal metabolic state, certain cancers utilize alternative pathways to obtain required nutrients. For example, macropinocytosis, an evolutionarily conserved endocytic pathway, functions as a feeding mechanism in cancer cells. Via macropinocytosis, extracellular proteins are engulfed into tumor cells, which can be proteolytically degraded further to free amino acids including glutamine, which enters the central carbon metabolic pathway to support tumor cell proliferation and survival [27, 42]. Other than extracellular protein, recent works from Chen and his group have shown how tumor cells in many cancer types can also internalize eATP via macropinocytosis to elevate intracellular ATP (iATP) levels by 50% to more than 100% in a few hours [41, 43]. Experimental evidence suggests that the increased iATP pool supports tumor cell proliferation and augments the survival of cancer cells under metabolic stress. Additionally, iATP content has been shown to increase drug resistance to multiple chemotherapeutic agents and tyrosine kinase target medications by upregulating the efflux pump activity of ABC transporters, phosphorylation of the PDGFR, and its associated Akt-mTOR and Raf-MEK pathways [44–46]. Interestingly, drug-resistant cancer cells exhibit even higher ATP levels than drug-sensitive cancer cells from which the resistant cells are derived [47, 48]. Moreover, macropinocytosis-mediated eATP internalization and its resultant elevated iATP levels have been shown to play a significant role in increasing migration and invasion by upregulating EMT-TFs and their associated activities [49].

### Extracellular ATP enhances cancer cell motility and invasion

Accumulating experimental evidence suggests that eATP, macropinocytosis, and purinergic receptors (both P2YRs and P2XRs) work together for activation of the EMT program and promote tumor cell migration and invasion. Recent work has shown that, in non-small cell lung cancer (NSCLC), elevated levels of iATP via macropinocytosis-mediated eATP internalization subsequently upregulates mesenchymal markers such as vimentin and EMT master transcription factors including SNAIL and ZEB1. These concertedly enhance the formation of filopodia and MMP enzyme secretion to promote cell migration and invasion. Moreover, knockout of the sorting nexin 5 (*SNX5*) gene (an essential gene for macropinocytosis) inhibits ATP internalization and suppresses cancer cell proliferation and cell migration in vitro and in vivo [49]. Recent work has also shown that eATP functions as a critical master inducer and regulator for EMT, in a manner similar to TGF-β at the level of transcription, translation, metabolism, and functionality in cancer cells. Specifically, RNAseq analysis indicates that eATP, like TGF-β, induces and regulates EMT associated-gene expression in lung cancer cells. Further analysis identified 11 genes that are consistently and significantly upregulated by either eATP or TGF-β at 2 and 6 h of treatment [50]. Some of these genes, such as *SOX8*, *BMP6*, *MMP10*, are well-known to play important roles in EMT, whereas the role of the remaining genes in their ability to induce EMT is either vaguely or completely unestablished. Furthermore, eATP treatment has also led to alterations in levels of metabolites in various metabolic pathways, including lactose synthesis and degradation, the Warburg effect, pyruvate metabolism, and oxidation of long-chain and branched-chain fatty acids [50]. This phenomenon is not surprising, as these pathways are also known to be related to and impacted by EMT programs. The same RNAseq analysis also identified 7 consistently and significantly downregulated genes by eATP [50]. Thus, the upregulation of these genes is likely to inhibit EMT and invasion. Moreover, metabolomics analyses demonstrate that the metabolic changes induced by eATP are involved in glycolysis, glutaminolysis, cytoskeleton remodeling, and the reactive oxygen species (ROS) pathway, which corresponds to the phenotypic changes associated with EMT and invasion [50]. What remains unclear is what alterations in gene expression and metabolic changes, relative to changes induced by TGF-β, may be at longer eATP treatment times, such as at 24 h, 48 h, or even longer. Further functional bioassays verified that among the 11 upregulated genes, one novel gene, *stannocalcin-1* (*STC1*), is involved in eATP-mediated cell proliferation, invasion, drug resistance, and EMT [51].

Recently, it has been reported that eATP also induces and regulates the formation of cancer stem cells (CSCs) [51]. In this study, it was found that eATP induces the expression of surface protein markers, CD44, CD166, and CD55 of CSCs. As a result, the CSCs subpopulation was increased by ~ 15–20% in treated A549 lung cancer cells. Furthermore, *stannocalcin-1* (*STC1*) [52–56] one of the 11 genes upregulated by eATP, was found to significantly contribute to A549 cells’ EMT and CSCs formation by upregulating mitochondrial ATP synthesis [51].

Another role of eATP is the modulation of cell motility and invasion as an extracellular signaling molecule. The increased level of eATP in the TME can act on purinergic (P2) receptors to promote cell migration and invasion. For example, in breast, cervical, and neuronal cell lines, ATP is released into the microenvironment in response to cell death modulated by chemotherapeutic agents. ATP subsequently activates downstream effectors such as p42/44 MAPK and p38 MAPK via P2X7 or P2Y12 signaling to upregulate inflammatory protein cyclooxygenase (COX-2) and enhance the activity of MMP-2 enzyme [57]. It is also noteworthy that ATP can act on P2X6 receptors to induce Ca^2+^ influx activity as well as regulate the phosphorylation of MAPK family ERK1/2 and modulate downstream effectors such as the expression on MMP9. This ATP-P2RX6-Ca^2+^-p-ERK1/2-MMP9 axis-promoted cell migration and invasion were observed in experimental models such as the renal cell carcinoma (RCC) cell line (i.e. RCC mouse model), as well as histological analyses of clinical samples [58]. Another study showed that ATP-modulated SOX-9 signaling via P2Y2 receptors is a crucial mechanism in mediating tumor metastasis [59]. In the breast cancer model, ATP mediates IL-6 release and activates JAK1-STAT3 signaling via P2Y2 receptors, upregulating SOX9 and its target gene CEACAM5/6, which is involved in tumor invasion [59]. In addition, direct activation of the P2Y2 receptor by ATP has been shown to activate the EMT process, which upregulates the transcription factor Snail and ILs while downregulating E-cadherin and claudin in prostate cancer cells [60]. Further analyses indicate that P2Y2R-silencing suppresses the EMT program, cell motility, and invasion both in vitro and in vivo [60]. Consistent with this finding, stimulation of the P2Y2 receptor with eATP promotes the EMT program, cancer cell migration, and invasion via activation of ERK1/2, p38, and EGFR signaling pathways in human PC-3,1E8 and 2B4 prostate carcinoma cell lines and human MDA-MB-231 breast cancer cells [61–63].

Additionally, eATP acting at the P2X7R has been implicated in the induction of EMT, promotion of MMP and cathepsin secretion, and the increased motility and invasive capacity in human melanoma, breast, and prostate cancer cells via activation of Ca^2+^-activated SK3 potassium channels or the PI3K/AKT pathway [64–66]. More intriguingly, recent work reveals that TGF-β causes release of ATP from human lung cancer cells (A549) via exocytosis. The exocytosed ATP then induces actin remodeling, such as the formation of actin stress fibers which are associated with cell migration, through the activation of P2X7 receptors. Knockdown of the P2X7 receptor or using P2 receptor inhibitors suppressed TGF-β-induced migration and actin remodeling [67]. Moreover, eATP-induced Ca^2+^ signaling through the P2Y11 receptor drives actin reorganization and filopodia formation and promotes cell migration in human hepatocellular cancer cells [68]. Thus, exocytosed ATP works in an autocrine and paracrine fashion to mediate EMT. The relationship between exocytosed ATP and macropinocytosis-internalized ATP is presently unclear.

**Fig. 2:**
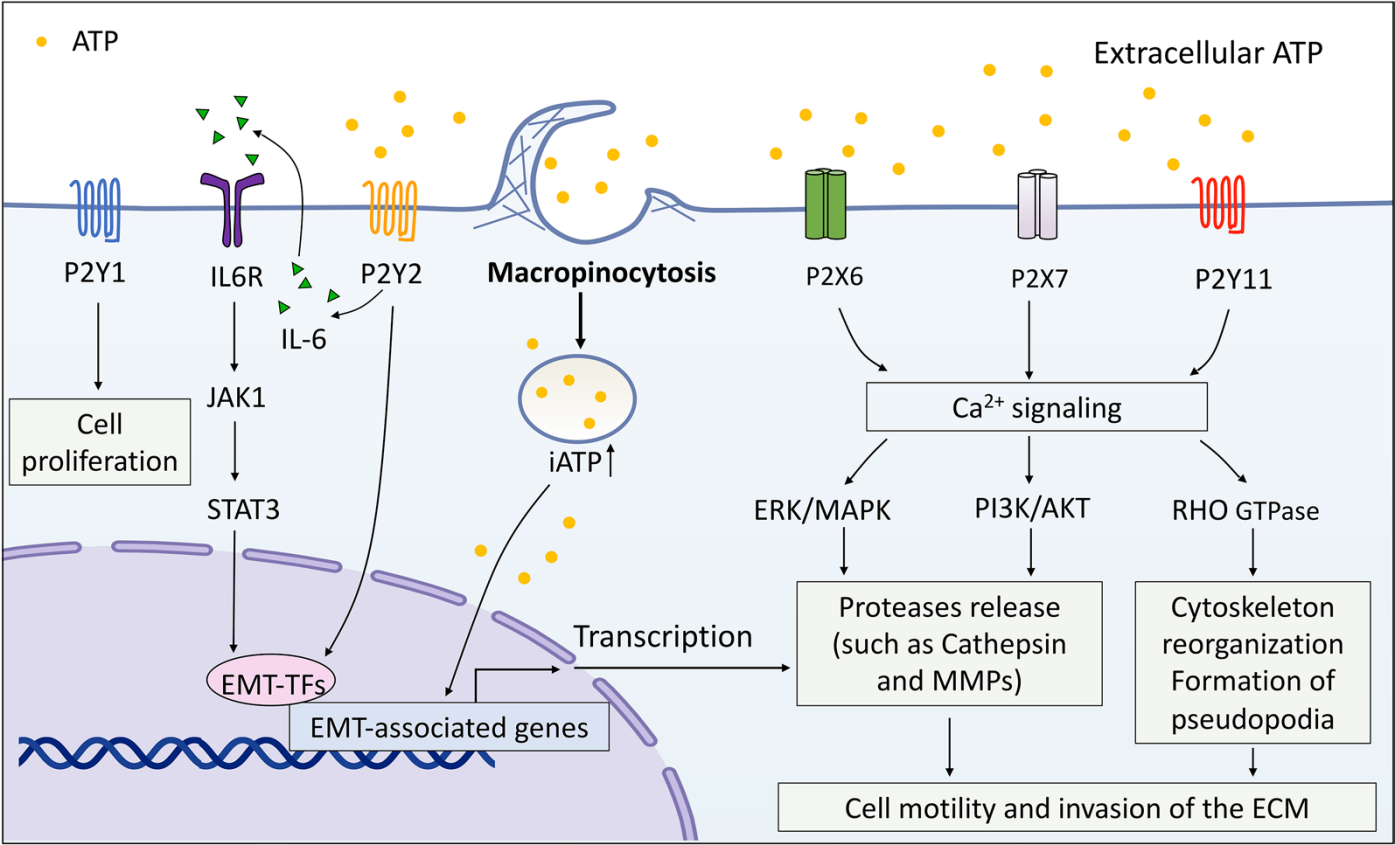
ATP’s roles in epithelial-mesenchymal transition and invasion. Extracellular ATP is internalized primarily by macropinocytosis to significantly elevate intracellular ATP levels and induce EMT progress through upregulation of mesenchymal markers and downregulation of epithelial markers via enhanced transcription factors such as Snail and Slug. Extracellular ATP also acts as a messenger molecule which binds and activates purinergic signaling via P2XRs or P2YRs. P2X6R, P2X7R or P2Y11R ligation by extracellular ATP promotes increases in intracellular Ca^2+^and activation of ERK/MAPK, PI3K/AKT, RHO GTPase. These pathways drive the release of proteases such as MMPs and cathpsins and regulate the formation of filopodia and other actin-rich structures supportive of cancer cell invasion into the ECM. P2Y1R and P2Y2R of cancer cells are also activated, thus promoting cancer cell proliferation and stimulation of EMT

These studies provide evidence to support the notion that eATP in the TME plays a crucial functional role in tumor migration and invasion, mainly through internalization by tumor cells via macropinocytosis or activation of P2XRs or P2YRs. ATP contributes to EMT induction and invasion in the following 3 aspects: (1) eATP acts as an extracellular messenger molecule by binding and activating various purinergic receptors, leading to PR-mediated specific signaling for EMT induction; (2) eATP is internalized via macropinocytosis and significantly enhances the iATP level in cancer cells. In turn, the elevated iATP reinforces the biochemical/enzymatic reaction driven by ATP, such as protein phosphorylation in signal transduction [43, 44, 49]; (3) ATP functions as a transcriptional cofactor involved in various inducing EMT-related genes and suppressing anti-EMT genes. In summary, all of these processes work together in the induction of EMT and the initiation of tumor invasion (Fig. 2).

### ATP in overcoming anoikis

**Fig. 3:**
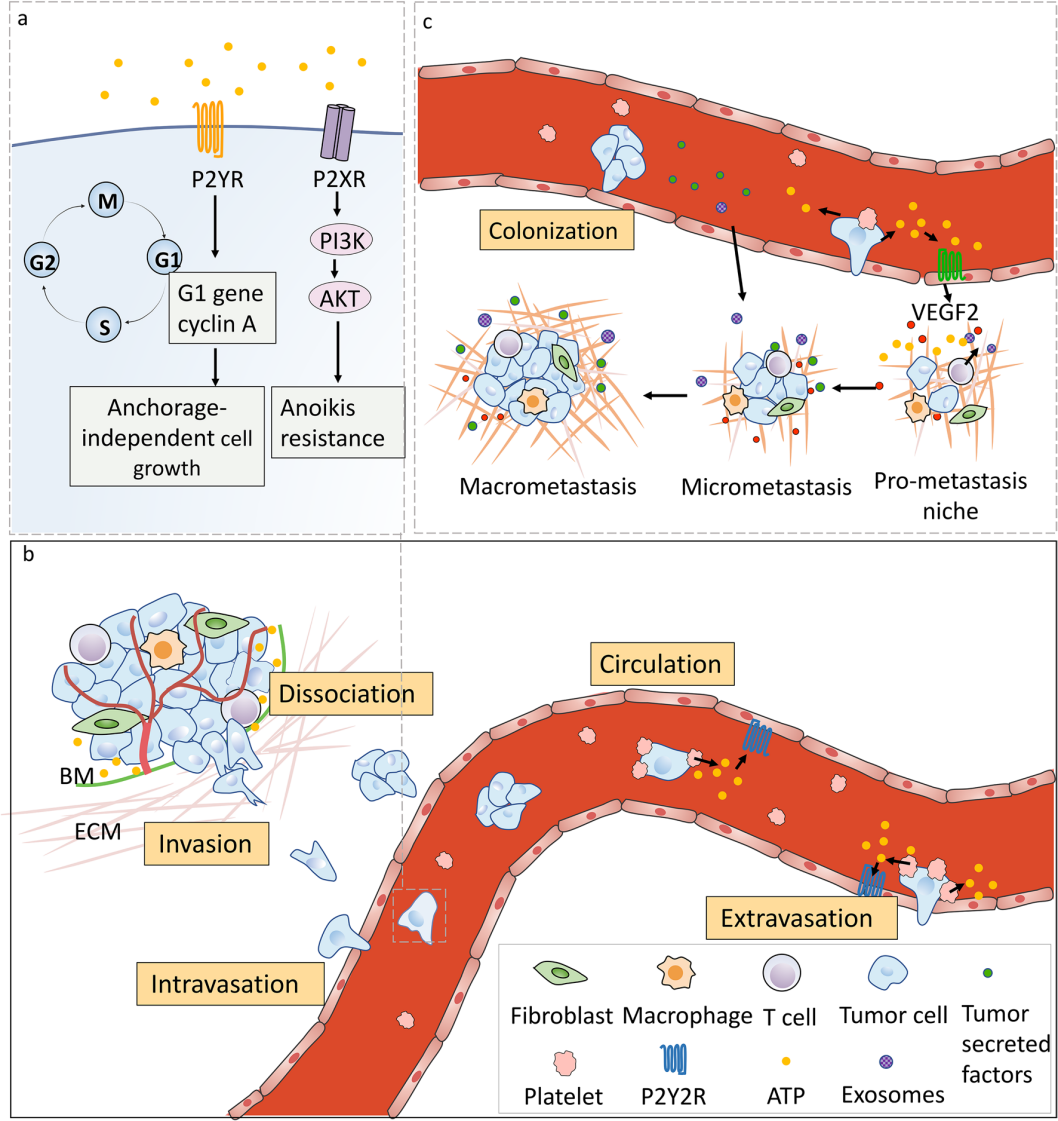
ATP’s roles in intravasation, circulation, extravasation and colonization. **a** Extracellular ATP’s role in overcoming anoikis. ATP induces the late G1 gene cyclin A expression and stimulates anchorage-independent cell growth by activating P2Y receptors. P2XR can also activate the stimulation of intracellular pathways, such as the PI3K/AKT pathways, that contribute to overcoming anoikis resistance. **b** Extracellular ATP plays a crucial role in platelet-mediated transmigration of tumor cells through the vascular endothelium. In the bloodstream, cancer cells rapidly associate with and are coated by the circulating platelets. This process triggers ATP release from platelets and facilitates tumor cell passage across the endothelial layer. More specifically, the ATP released from active platelet-activation of the P2Y2 receptor induces the endothelial cells to retract from each other, which promotes the opening of the endothelial barrier, thus facilitating cancer cell intravasation and extravasation. **c** During extravasation, extracellular ATP activates the P2Y1 receptor of vascular endothelial cells, and induces the releases of VEGF2 to initiate angiogenesis in the metastatic niche. Extracellular ATP acts on P2X7 receptors of immune cells to stimulate the release of plasma membrane-derived microvesicles and exosomes. These vesicles may potentially impact the formation of the pre-metastatic niche and lead to an increased capacity for metastatic outgrowth

It is important to note that before cancer cells are able to metastasize, they must first overcome anoikis, a form of programmed cell death that occurs when anchorage-dependent cells detach from the surrounding ECM, thus disrupting integrin ligation [69]. Under normal conditions, when integrins on the cell surface are in contact with the ECM, focal adhesion kinase (FAK) is activated by phosphorylation, which in turn triggers a phosphorylation cascade ending with the activation of Akt, thus promoting cell survival. If the integrin on the cell’s surface detaches from the ECM, the cell survival signals cease, leaving pro-apoptotic proteins such as Bax and Bad uninhibited and ultimately leading to initiation of cell death [70]. Cancer cells can overcome and override this important negative switch in a variety of ways. Namely, eATP is capable of promoting anoikis resistance and migration, a prerequisite for metastasis. For instance, ATP induces expression of the late G1 gene cyclin A and stimulates anchorage-independent cell growth [71]. Recent work has shown that eATP stimulates the activation of PI3K/Akt through purinergic receptors, promoting anoikis resistance and metastasis in osteosarcoma, hepatocellular carcinoma, and prostate cancer (Fig. 3a).

### ATP in intravasation, circulation and extravasation

Once a carcinoma cell successfully invades the peritumoral tissue, it can intravasate into blood vessel or lymph vessel circulation. This process by which cancer cells cross the endothelial wall is known as transendothelial migration (TEM). Recent works unveil a crucial role for ATP in platelet-mediated TEM. Once in the bloodstream, cancer cells rapidly associate with and become coated by the circulating platelets. This process is triggered by the tissue factor expressed on the tumor cell and leads to the formation of thrombin and the activation of platelets [72]. Schumacher and colleagues demonstrated that co-incubation of tumor cells with platelets triggers ATP release from platelets and facilitates tumor cell passage across the endothelial layer by activation of the P2Y2 receptor (Fig. 3b) [73]. Specifically, ATP released from activated platelets induces the endothelial cells to retract from each other, which enhances the permeability of the vasculature capillary walls, thus facilitating cancer cell intravasation and extravasation [72, 74–76]. Moreover, eATP enhances the expression of adhesion molecules on the endothelium and promotes the adherence and arrest of tumor-platelet emboli to the endothelium of the vascular wall [77, 78]. In another study, it was shown that the release of ATP from necroptotic endothelial cells promotes the opening of the endothelial barrier, thereby facilitating extravasation [79].

During circulation, tumor cells are exposed to various biological and mechanical stressors that may lead to cell death, including detachment from the ECM, attack by the immune system, and exposure to mechanical force [72, 80]. Mechanical forces such as fluid shear stress (FSS) and mechanical deformation in the microvasculature are responsible for a considerable loss of tumor cells [81]. Recent work has shown that tumor cell-released ATP can protect the cell from lethal mechanical injury. In highly metastatic breast cancer cell lines, the membrane stretches endured in microcirculation induce ATP release from mechanosensitive PANX1 channels. The released ATP then acts in an autocrine fashion, promoting survival signaling via P2Y-purinergic receptors and eventually protecting tumor cells against mechanical stress-induced cell death [82].

### ATP in metastatic colonization

To successfully proliferate and form a metastatic colony in distant tissue, disseminating tumor cells must possess two qualities: (1) tumor-initiating ability and (2) adaptive programs to acclimatize the parenchymal tissues or shape the microenvironment in the secondary site [83]. Experimental evidence indicates that eATP influences the ability of disseminating tumor cells to proliferate and thereby initiate metastatic outgrowth in secondary tissues. Extracellular ATP has been shown to stimulate the release of plasma membrane-derived microvesicles and exosomes from immune cells in the stroma via activation of P2X7 receptors. The exosomes (or microvesicles) contain various bioactive compounds including IL-1beta, microRNAs, tissue factors, proteases, etc. [84–86]. These vesicles can travel through the circulatory system and are deposited in distant tissues, which may potentially trigger the formation of the pre-metastatic niche and lead to an increased capacity for metastatic outgrowth. Moreover, during extravasation, eATP activates the P2Y1 receptor on vascular endothelial cells to induce the release of VEGF2 to initiate angiogenesis in the metastatic niche in order to supply more oxygen and nutrients, ultimately promoting tumor cell proliferation (Fig. 3c).

## Strategies for targeting ATP involved metastasis

Metastasis, or the consequences of its ineffective treatment, is a major contributor to death among cancer patients. Cancer patients initially diagnosed with metastatic disease have a much lower survival rate than patients diagnosed with localized disease. Currently, a large number of anti-metastatic agents, as well as novel, more advanced immunotherapy, are available to prevent and treat metastases. For instance, multiple FDA-approved drugs such as denosumab, bevacizumab, and saracatenib show a clinical effect on the prevention of certain advanced or metastatic cancers. Moreover, immunotherapy (for example, programmed cell death protein 1 [PD1] and cytotoxic T-lymphocyte-associated antigen 4 [CTLA-4] immune checkpoint inhibitors) has resulted in significantly improved long-term survival in patients with metastatic melanoma, renal cancer, non-small cell lung cancer, and squamous cell carcinoma [87, 88].

**Fig. 4:**
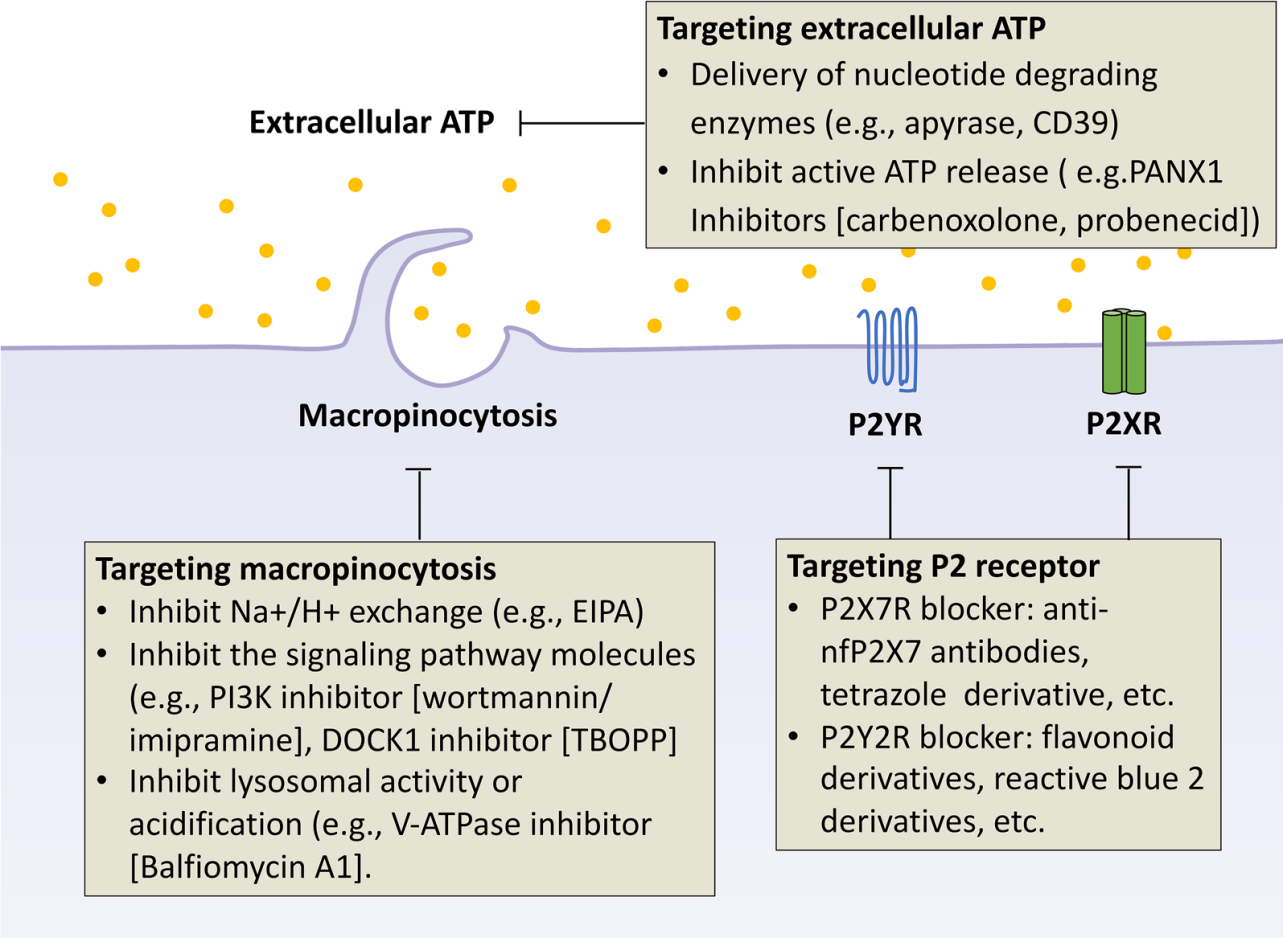
The strategies for targeting ATP-involved metastasis. These strategies include targeting extracellular ATP in the TME and blocking macropinocytosis or P2 receptors. Delivery of the inhibitors of ATP synthesis or nucleotide degrading enzymes (for example, apyrase) in tumor tissues via pH-sensitive or ATP-sensitive NPs provides a potential strategy to reduce ATP levels in the TME. Moreover, blockade of ATP-release via the PANX1 channel has been proposed to minimize metastasizing carcinomas and inhibit metastasis. In addition, targeting macropinocytosis or purinergic receptors by various small molecules or biologics are considered to be effective strategies to suppress tumor growth and metastasis

In light of intratumoral eATP’s ability to enhance the metastatic potential of cancer cells through different mechanisms, the development of treatment strategies to target eATP and its associated pathways is both reasonable and promising. At least three approaches may be worthy of pursuit: eliminating eATP in the TME, blocking macropinocytosis, and/or selectively targeting P2 receptors (Fig. 4). An overview of several pharmaceutical small molecules or biologics targeting eATP, macropinocytosis, or P2 receptors are listed in Tables 1, 2, 3.

### Targeting extracellular ATP

As discussed extensively above, high intratumoral extracellular ATP (ieATP) levels in the TME facilitate tumor growth, drug resistance, and metastasis. Therefore, targeting ieATP itself in the TME has been considered a novel anticancer therapy. For example, the development of nanobodies provides a potential vehicle and selective tool to reduce ATP levels in the TME. Specifically, inhibitors of ATP synthesis or nucleotide degrading enzymes (for example, apyrase) could be specifically delivered to tumor tissues by using a nanobody-based delivery system [89, 90]. As an example, CD39, an ATP-diphosphohydrolase, rapidly and preferentially metabolizes ATP or ADT to AMP; thus, local application of the recombinant soluble form of human CD39 might decrease ATP levels in the TME [91].

Furthermore, the TME is an acidic environment due to glycolytic cancer cell metabolism, hypoxia, and insufficient blood perfusion [92]. Thus, pH-sensitive nanoparticles (NPs) could prove an effective method of delivery of compounds that target eATP in the vicinity of cancer cells [93].

Moreover, inhibition of ATP release into the TME may lead to a decreased capacity for metastasis. In particular, the pannexin 1 channels or P2X7R would serve as a suitable target for ATP secretion. Furlow et al. reported that ATP secreted by breast cancer cells through the PANX1 channel could act as a suppressor of apoptosis via P2Y purinergic receptors and thus permit tumor cells to survive in the microvasculature and promote metastatic spreading. The authors also indicated that inhibiting PANX1 by using non-selective PANX1 inhibitors (such as carbenoxolone or probenecid) efficiently increased cell death and reduced secondary lung metastasis in a mouse model (Table 1). For this reason, the blockade of ATP release via the PANX1 channel has been proposed to inhibit metastasizing carcinomas [82].

**Table 1:** Small molecule inhibitors and biologics used to target extracellular ATP

Inhibitor	Target (s)	Mechanism of action	Development stage	Indications	Refs.
Apyrase (e.g., CD39)	Extracellular ATP	Degrade extracellular ATP to adenosine	Preclinical	Not available (N/A)	[94]
Carbenoxolone	Non-selective PANX1 inhibitor	Inhibit ATP secretion	In the clinic	Peptic, esophageal and oral ulceration and inflammation	[82]
Probenecid	Non-selective PANX1 inhibitor	Inhibit ATP secretion	In the clinic	Chronic gout and gouty arthritis	[82]

### Targeting macropinocytosis

Blocking macropinocytosis proves another suitable candidate for anti-cancer and anti-metastatic therapy. Macropinocytosis has been demonstrated to be exploited by carcinoma cells to replenish scarce energy and nutrients for sustained rapid proliferation [42, 95]. In particular, Ras-transformed cancer cells utilize macropinocytosis to internalize extracellular proteins and then degrade them to amino acids such as glutamine, which provides a key source for carbon metabolism and cell proliferation in low nutrient environments [42]. Also, recent studies revealed that cancer cells internalize eATP to promote cancer cell growth, drug resistance, EMT induction, tumor invasion, and cancer stem cell formation [41, 43, 44, 49, 96–98]. Moreover, cancer cells have also been shown to internalize secreted exosomes, which shape the TME and facilitate angiogenesis, metastasis, and immunosuppression [99].

For these reasons, disrupting macropinocytosis is a potential strategy for targeting and inhibiting metastasis. Several different strategies are currently under investigation, including: (1) inhibition of Na^ +^ /H^+^ exchange; (2) inhibition of the signaling pathway molecules involved in macropinocytosis activity; (3) inhibition of lysosomal activity or acidification. For instance, studies revealed that EIPA (5-[N-ethyl-N-isopropyl] amiloride) blocks macropinocytosis and actin polymerization by inhibiting the Na^+^ /H^+^ exchanger (NHE). Targeting macropinocytosis by interrupting the signaling pathway network regulators is also considered effective. It is reported that the phosphoinositide-3-kinase (PI3K) inhibitor, wortmannin, blocks the scission of macropinosomes from the cell surface, thereby suppressing cell motility, invasion, and metastasis in pancreatic cells and nude mice [100, 101]. In addition, 1-[2-(3′-(trifluoromethyl)-(1,1′-biphenyl)-4-yl)-2-oxoethyl]-5-pyrrolidinylsulfonyl-2 (1H)-pyridone (TBOPP), a selective inhibitor of Dedicator of cytokinesis 1(DOCK1), inhibits macropinocytosis and invasion in cancer cells and also suppresses cancer growth and metastasis in vivo [102]. Lin and colleagues have screened FDA-approved macropinocytosis inhibitors and found that the optimal compound (imipramine) inhibits macropinocytosis and could be a suitable candidate as a therapeutic agent in pathological processes involving macropinocytosis [103].

Additionally, targeting lysosomal acidification and their acidic pH can indirectly block macropinocytosis and protein degradation. It has been recently reported that vacuolar-type H^+^-ATPases (V-ATPases) inhibitors, such as Balfiomycin A1, or chloroquine analogs, such as hydroxychloroquine (HCQ), can indirectly inhibit macropinocytosis in tumor cells by interfering with lysosomal acidification (Table 2) [104].

**Table 2:** Small molecule inhibitors and biologics used to target macropinocytosis

Inhibitor	Target (s)	Mechanism of action	Development stage	Indications	Refs.
EIPA	Na^+^/H^+^ exchange	Impact on submembranous alkaline pH	Preclinical	Not available (N/A)	[106, 107]
Gefitinib	EGFR	Inhibit the macropinocytosis pathway	FDA Approval, Phase 2 (completed, NCT02804776)	Non-small cell lung Cancer, Non-small cell lung Cancer	[108]
GCS-100	Galectin-3	Inhibit the macropinocytosis pathway	Phase 2 (completed)	Chronic kidney disease	[109, 110]
TBOPP	DOCK1	Repress DOCK1-mediated macropinocytosis	Preclinical	N/A	[102]
Wortmannin, LY294002	PI3K	Inhibit PI3K signaling pathway	Preclinical	N/A	[100, 111]
Torin1 and AZD2014	mTOR	Suppress proteins scavenging	Preclinical	N/A	[112, 113]
Sepantronium bromide (YM155)	AMPK	Block mTORC1	Phase 2 (completed)	Non-Hodgkin’s lymphoma	[105, 114]
Blebbstatin	myosin II	Blocks the myosin heads in a products complex with low actin affinity	Preclinical	N/A	[115]
Cytochalasin D	actin	Inhibit actin polymerization inhibits both the rate of actin polymerization and the interaction of actin filaments in solution	Preclinical	N/A	[116, 117]
IPA-3	Pak1	Impact on actin polymerization	Preclinical	N/A	[118, 119]
Bafilomycin A1	v-ATPase	Impact on lysosomal acidic pH	Preclinical	N/A	[120]
Hydroxychloroquine (HCQ)	Lysosomal	Inhibits lysosomal acidification	In the clinic, Phase 1 (completed)	Antiphospholipid Syndrome, Multiple Myeloma	[121]
GNS561	Lysosomal	Suppress lysosomal activity	Phase 1 (completed)	Primary and Secondary liver cancer	[122]

In conclusion, inhibition of macropinocytosis by multiple approaches demonstrates an effective block of the internalization of extracellular proteins and eATP into tumor cells. Some of these approaches may potentially be effective therapies for suppressing tumor growth and metastasis. It is important to test these approaches in vivo and examine their safety and efficacy in clinical trials. In particular, several macropinocytosis inhibitors such as GCS-100, sepantronium bromide (YM155), hydroxychloroquine (HCQ), and GNS561 have entered the clinical setting. Of note, gefitinib has already been approved by the FDA for the treatment of patients with lung cancer. In addition, a phase II study evaluating the safety and tolerability of sepantronium bromide (YM155) in combination with rituximab showed encouraging antitumor activity and durable responses in patients with non-Hodgkin lymphoma [105] (Table 2).

### Targeting P2 receptors

Recent evidence shows that purinergic receptors also play an important functional role in tumor invasion, intravasation, extravasation, and metastasis. Among the P2R family, P2X7R, P2Y2R, and P2Y11R are promising targets for anti-metastatic therapy. For example, activation of the P2X7 receptor by high levels of ATP can promote tumor growth and cancer cell dissemination both in vitro and in vivo. Thus, several P2X7R blockers have been developed, which have been shown to inhibit P2X7 receptor-mediated activities and the invasive capability of tumor cells in experimental tumor models [65, 123, 124]. P2X7R competitive antagonists (such as suramin, suramin-like derivatives, or ATP derivatives) in their early development were small molecules expressing several negative charges that are capable of interacting with the positively charged residues within the ATP-binding cavity and compete for ATP-mediated P2X activation. Subsequently, other inhibitors (for example, Brilliant Blue G, AZ-11645373, CE-224,535, AFC-5128, JNJ-47965567) have been identified and developed to show increased specificity and selectivity for P2X7R (Table 3). These inhibitors targeted allosteric binding pockets other than the ATP-binding cavity, changing the conformation of or stabilizing the P2X tridimensional arrangement, and thus preventing ATP-mediated subunit rotation and turret closure (Table 3) [125, 126]. More recent studies have shown that polyclonal antibodies with a specific target on epitopes (E200) of nonfunctional forms of the P2X7 receptor have been used to treat skin cancer. The 200–216 amino acid sequence (E200) epitope is only present on nfP2X7 in tumor cells, but not on normal cells, such that the antibodies can specifically target cancer cells [125]. Now, a completed phase I clinical trial suggests that anti-nfP2X7 antibodies (BIL-010t) are a novel and safe therapy for basal cell carcinoma [127, 128].

**Table 3:** Small molecule inhibitors and biologics used to target P2 receptors

Inhibitor	Target (s)	Mechanism of action	Development stage	Indications	Refs
Suramin	P2X7/P2Y11 receptor orthosteric site	Binding to orthosteric site to compete ATP mediated P2X7 activation	In the clinic, Phase 3 (completed), Phase 2 (completed)	African sleeping sickness, Stage IV prostate cancer, Stage IIIB-IV breast cancer	[131]
NF279 (Suramin analog)	P2X7 receptor orthosteric site	Binding to orthosteric site to compete ATP mediated P2X7 activation	Preclinical	Not available (N/A)	[132]
2, 3-dialdehyde ATP (oxidized ATP)	P2X7 receptor orthosteric site	Binding to orthosteric site to compete ATP mediated P2X7 activation	Preclinical	N/A	[125, 133, 134]
Brilliant Blue G (BBG)	P2X7 receptor inter-subunit allosteric pocket	Binding to the inter-subunit allosteric pocket preventing ATP induced rotation of each subunit and closure of the turret	Preclinical	N/A	[135]
AZ-11645373	P2X7 receptor inter-subunit allosteric pocket	Binding to the inter-subunit allosteric pocket preventing ATP induced rotation of each subunit and closure of the turret	Preclinical	N/A	[136, 137]
JNJ-47965567	P2X7 receptor inter-subunit allosteric pocket	Binding to the inter-subunit allosteric pocket preventing ATP induced rotation of each subunit and closure of the turret	Preclinical	N/A	[138, 139]
CE-224,535	non-competitive antagonist of the human P2X7 receptor	Purinergic P2X7 receptor antagonists	Phase 2 (completed)	Rheumatoid arthritis	[140]
AFC-5128	noncompetitive, negative allosteric modulators	Purinergic P2X7 receptor antagonists	Preclinical	N/A	[138, 141]
BIL-010t	E200 peptide in P2X7 extracellular domain	Polyclonal antibodies with a specific target on epitope (E200) of nonfunctional forms of the P2X7 receptor to block P2X7 activity	Phase 1 (completed)	Basal cell carcinoma (BCC)	[127]
BIL06v	E200 peptide in P2X7 extracellular domain	Peptide-protein conjugate vaccine based on the E200 sequence	Phase 1 (completed)	Solid tumors	[142]
AR-C126313	Selective and competitive P2Y2 receptor antagonist	Inhibited responses mediated by endogenous P2Y2 receptors	Preclinical	N/A	[143]
AR-C118925XX	Selective and competitive P2Y2 receptor antagonist	Iinhibited responses mediated by endogenous P2Y2 receptors	Preclinical	N/A	[144, 145]
Reactive Blue-2	Selective antagonist at P2Y receptors (P2Y2 / P2Y11)	Inhibited responses mediated by endogenous P2Y2 receptors	Preclinical	N/A	[146]
Flavonoid	Selective antagonist at P2Y receptors	Reduce the amplitude of the P2Y2 receptor response to UTP	Phase II/ III	Hematopoietic/lymphoid or solid cancer	[146, 147]
NF157	Highly selective nanomolar P2Y11 antagonist	Competitive antagonism against ATP	Preclinical	N/A	[148, 149]
NF340	Highly selective nanomolar P2Y11 antagonist	Competitive antagonism against ATP	Preclinical	N/A	[148]

In addition to P2Y2R’s function in promoting cell invasion and metastasis, it has also been shown to play a predominant role in platelet-mediated tumor cell trans-endothelial migration, which supports cancer cell extravasation [129]. Several P2Y2 receptor antagonists including AR-C118925XX, flavonoid derivatives, and reactive blue 2 derivatives have been shown to exhibit the ability to block P2Y2 activation [130]. Some experimental evidence has suggested that pharmacological blocking or genetic silencing of P2Y2R inhibited platelet-mediated inter-endothelial junctions from opening and suppressed tumor cell extravasation. In addition, P2Y2-deficient mice showed a dramatic reduction in cancer cell dissemination [73].

In summary, P2X or P2Y receptors appears to be a promising target for reducing metastatic dissemination since it is deeply involved in the ATP-induced metastasis cascade. As multiple P2X and P2Y receptors are involved in these processes, blocking specific purinergic receptors may be effective in combating eATP-mediated tumorigenesis and metastasis. However, specifically targeting cancer P2X or P2Y receptors without blocking their signaling in normal cells may be a challenge.

## Conclusions and future perspectives

Metastasis is considered a multi-step process, and the EMT program is crucial to the invasion and metastatic spread of most cancer types. Accumulating evidence in recent works shows the importance of EMT-related changes in many phases of metastasis. Among them, ATP, one of the major biochemical components in the TME, has emerged as a critical molecule that affects tumor growth and metastatic dissemination. In this review, we summarize the role of extracellular ATP in EMT, its critical function in each step of the invasion-metastasis cascade, and provide an organized description of the mechanisms of macropinocytosis and P2 receptor-mediated ATP-induced EMT and metastasis. As shown in Fig. 2, eATP is directly internalized into cancer cells via macropinocytosis, which increases the iATP levels, promotes the activity of EMT-related transcription factors snail and slug, and leads to an increased capacity for migration and invasion. It is possible that internalized ATP reinforces the EMT-related signaling pathway by upregulating phosphorylation of protein factors involved in EMT signaling, or by directly providing energy for cell movement during invasion. Moreover, eATP acts on several P2Y or P2X receptors that regulate various signaling pathways involved in activating EMT programs and inducing tumor invasion. Despite the growing number of studies accumulated over the past few decades investigating ATPs involvement in cancer metastatic, many questions remain to be answered. For example, how does internalized ATP contribute to cellular dynamics during tumor cell migration? What ATP-induced signaling pathway endows the most robust pro-metastatic properties to tumor cells? How is ATP involved in the late stages of metastasis, including mesenchymal-epithelial transition (MET), colonization, and the formation of the metastatic niche in a distant organ? Hence, a more detailed understanding of these mechanisms will be necessary to identify therapeutic targets and develop effective anti-metastatic therapies in the near future.

As discussed, eATP activates the EMT program and induces cell invasion via macropinocytosis and P2 receptor signaling. Thus, targeting P2 receptors and/or macropinocytosis has become a promising anti-metastatic therapeutic strategy, and substantial efforts have been made to develop antagonists of these pathways. In particular, various macropinocytosis inhibitors and P2R inhibitors have been tested in preclinical models and have shown remarkable anticancer effects without substantial side effects. However, very few of them have been evaluated in clinical trials in the treatment of patients with cancer. Thus, therapeutic agents targeting macropinocytosis or P2R need to be designed and tested both in preclinical and clinical settings. We believe further studies in these directions will drive innovative, safe, and effective therapies into the clinical setting. These novel therapies are likely to be beneficial for decelerating or preventing metastasis in cancer patients.

In conclusion, ample experimental evidence demonstrates that eATP is deeply involved in and actively contributes to almost every step of EMT and CSC formation and growth, both of which are necessary changes in order for metastasis to occur. Going one step further, it is conceivable that eATP, as an energy source, a signaling molecule, and a transcription cofactor, is highly likely to also be involved in later steps of metastasis such as intravasation, circulation, extravasation, MET, and colonization. More studies should target and be conducted in these areas for a clearer and fuller understanding of not only the overall roles of eATP in cancer, but also how to effectively and safely inhibit eATP’s functions to slow down or even halt metastasis to reduce cancer-related death.

## Data Availability

All data and materials are included in this published article.

## Abbreviations

eATP: Extracellular ATP
TME: Tumor microenvironment
ECM: Extracellular matrix
EMT: Epithelial-mesenchymal transition
MET: Mesenchymal-epithelial transition
ABC: ATP-binding cassette
CFTR: Cystic fibrosis transmembrane conductance regulator
MDR: Multidrug resistance
SUR: Sulfonylurea receptor
P2X7R: P2X purinergic receptor 7
PNAX1: Pannexin 1
NSCLC: Non-small cell lung cancer
oATP: Oxidized ATP
PR: Purinergic receptor
SNX5: Sorting nexin 5
TF: Transcription factor
MMP: Matrix metalloproteinases
CSCs: Cancer stem cells
FAK: Focal adhesion kinase
